# Contrasting responses of Central Asian rock glaciers to global warming

**DOI:** 10.1038/srep08228

**Published:** 2015-02-06

**Authors:** Annina Sorg, Andreas Kääb, Andrea Roesch, Christof Bigler, Markus Stoffel

**Affiliations:** 1 Climatic Change and Climate Impacts Group, Institute for Environmental Sciences (ISE), University of Geneva, Route de Drize 7, CH-1227 Carouge, Switzerland; 2 dendrolab.ch, Institute of Geological Sciences, University of Berne, Baltzerstrasse 1 + 3, CH-3012 Berne, Switzerland; 3 Department of Geosciences, University of Oslo, P.O. Box 1047, N-0316 Oslo, Norway; 4 Forest Ecology, Institute of Terrestrial Ecosystems, Department of Environmental Systems Science, ETH Zurich, Universitätstrasse 16, CH-8092 Zürich, Switzerland; 5 Department of Earth Sciences, University of Geneva, Rue des Maraîchers 13, CH-1211 Geneva 4, Switzerland

## Abstract

While the responses of Tien Shan glaciers – and glaciers elsewhere – to climatic changes are becoming increasingly well understood, this is less the case for permafrost in general and for rock glaciers in particular. We use a novel approach to describe the climate sensitivity of rock glaciers and to reconstruct periods of high and low rock glacier activity in the Tien Shan since 1895. Using more than 1500 growth anomalies from 280 trees growing on rock glacier bodies, repeat aerial photography from Soviet archives and high-resolution satellite imagery, we present here the world's longest record of rock glacier movements. We also demonstrate that the rock glaciers exhibit synchronous periods of activity at decadal timescales. Despite the complex energy-balance processes on rock glaciers, periods of enhanced activity coincide with warm summers, and the annual mass balance of Tuyuksu glacier fluctuates asynchronously with rock glacier activity. At multi-decadal timescales, however, the investigated rock glaciers exhibit site-specific trends reflecting different stages of inactivation, seemingly in response to the strong increase in air temperature since the 1970s.

As a result of increasing air temperatures, glaciers in the arid Central Asian Tien Shan ranges have experienced strong mass loss over the past decades^1^,^2^, with possible impacts on freshwater availability during the drier summer months. Ice-rich permafrost bodies could gain relative importance for water supply in future summers, since they are assumed to store a similar amount of freshwater as the glaciers in the Tien Shan do^3^,^4^. A particularly important role in the regional hydrological cycle is hereby played by rock glaciers, i.e. frozen debris lobes that creep downslope under gravity^5^ and typically contain between 20 and 80 vol% of ice^6^. However, estimates on the ice content stored in rock glaciers are highly variable and uncertain^7^, and seasonal water releases from rock glaciers are generally lower and tend to fluctuate less due to their insulating debris layer^8^,^9^.

Rock glaciers are a widespread permafrost phenomenon in the Tien Shan; in northern Tien Shan alone (approximately 5'000 km^2^), more than 1'000 rock glaciers have been inventoried^10^,^11^. Knowledge about the response of rock glaciers to climate change is thus of great interest, but so far, only few studies have investigated the link between climate and rock glaciers in general and in the Tien Shan region in particular^3^,^6^,^10^,^12^. Geothermal observations at Zhusalykezen pass (3'337 m a.s.l.) in Kazakhstan have revealed an average recent rise in permafrost temperature by 0.1 to 0.2°C a^−1^ at depths of 10 and 25 m, respectively, over the period 1974–2004^6^. Measurements of rock glacier front positions in the Tien Shan date back to 1923^13^ and exhibit high mean frontal advance rates of up to several meters per year, with an accelerating trend since the mid-1980s^3^,^10^. This is in line with observations of rock glaciers in the European Alps, where a number of them have significantly accelerated since the 1980s, presumably due to a range of reasons, among them increasing permafrost temperatures^14^. Synchronicity of rock glacier activity as observed in the Alps^14^, however, has not yet been assessed in the Tien Shan, where knowledge on the long-term climatic impacts on rock glaciers remains highly fragmental.

Here we extend the current state of knowledge on rock glacier activity in the Tien Shan in the temporal and spatial dimensions, and present the world's longest record of annual rock glacier activity. By combining growth anomalies in trees growing on rock glaciers, repeat aerial photographs and high-resolution satellite imagery, we reconstruct the activity of four rock glaciers in the Kyrgyz and Kazakh parts of the Tien Shan range over the past 120 years. The aims were to assess relations between the activity of these rock glaciers and climatic variations, to test for regionally synchronous periods of high and low rock glacier activity in the Tien Shan, and to decipher their possible climate sensitivity and development status.

As a peculiarity in regard to many other periglacial regions, the investigated rock glaciers reach down below the tree line (Fig. 1, Table S1). The rock glacier fronts are located close to the zero-degree-isotherm of mean annual air temperature at around 2'700 m a.s.l.^6^, i.e. at elevations where permafrost does not usually occur outside the rock glaciers themselves^11^ and where the rock glaciers are probably only slightly below the freezing point^7^. As a consequence, these bodies exhibit comparably low viscosity, fast movement rates and a potentially particular sensitivity even to minor changes in surface energy balance and ground temperatures^3^,^6^. The steep terminal fronts (>37°) with loose boulders and “drunken trees” growing on the rock glaciers indirectly confirm the high activity of the investigated rock glaciers^5^ (Figs. S1–2).

The approach presented here capitalizes the fact that trees growing on rock glaciers suffer from enhanced rock glacier activity. Horizontal and vertical movements are recorded in the growth-ring series of trees and can thus be used as a proxy of rock glacier activity with annual resolution. Based on the tree-ring records of 250 Tien Shan spruces (*Picea* *shrenkiana*) and 30 junipers (*Juniperus* sp.) growing on the rock glaciers investigated in this study, we document more than 1500 growth anomalies induced by past rock glacier movements (Tables S2 and S3, Fig. S3). We use the annual ratio between the number of reacting and the total number of sampled trees (I_t_ index^15^; Figs. S4–7) as a proxy of rock glacier activity. Effects of summer air temperature on rock glacier activity from 1895 to 2011 are quantified using distributed lag models, which account for lagged effects of air temperature over several years^16^. The results were then complemented with displacement rates derived from image cross-correlation^17^,^18^ of repeat aerial photographs from Soviet archives dating back to the year 1943, declassified CORONA satellite images of around 1970, and high-resolution, present-day satellite images (Table S4).

## Results and Discussion

At decadal timescales, the frontal zones of the investigated rock glaciers show a common signal of enhanced and reduced activity since 1895, and reveal particularly active periods in the 1940s and 1990s (Fig. S8, Animation S1). The synchronous behavior of the rock glacier movements suggests that internal and topographic characteristics are superimposed by an external driver, decadal climate variations in the present case^19^. Increasing rock glacier surface speed is largely a consequence of rising ground temperatures, which decrease the viscosity of the rock glacier ice core^14^. However, the effect of climatic changes on the different energy balance components and eventually on ground temperature involves complex processes and interactions, most importantly related to snow cover timing and thickness, and energy exchange within the blocky surface layer^20^,^21^. In spite of the complex coupling between climatic variations and ground ice temperatures, rock glacier activity has previously been shown to increase in comparably direct response to warm summers in a number of cases^14^,^22^,^23^. Such immediate coupling between the atmosphere and movement of – thermally inert – permafrost could be fostered by increasing lateral or vertical water percolation during warm summers, for instance from increased ice or snow melt, through related heat advection or even hydraulic mechanisms.

**Figure 2:**
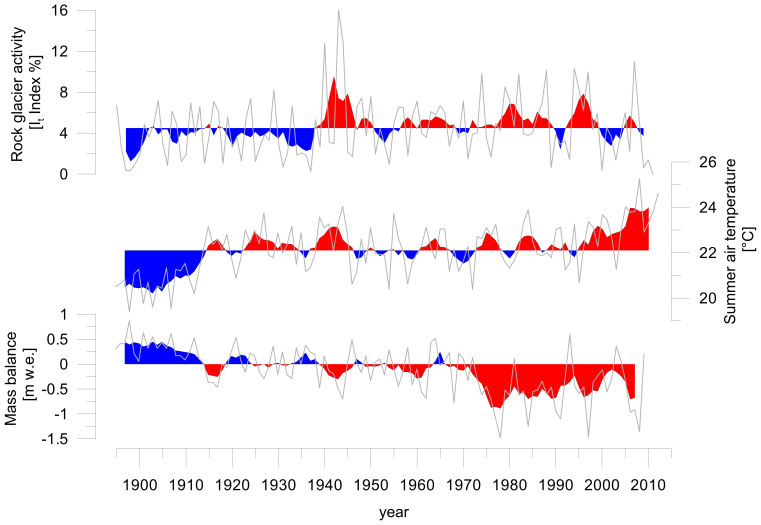
Periods of high rock glacier activity correspond well with periods of warm summers and negative mass balance at Tuyuksu glacier (1895–2011). Above and below average 5-year-running-means are indicated in red and blue, respectively; annual data is shown in grey. Rock glacier activity is shown as the mean I_t_ index of Karakorum, Ordzhonikidze and Turgen Aksu rock glaciers (Fig. S8). Summer air temperature is the mean of June-August air temperatures in Almaty (Fig. 1). Mass balance of Tuyuksu glacier is in meters water equivalents (m w.e.). Data sources: Supplementary Information.

The previously observed high correlation of rock glacier surface speed and variations in air temperature^23^,^24^ is confirmed by our results: we find that increasing summer (JJA) air temperatures correlate with increasing rock glacier activity at annual (Fig. S9) and decadal timescales (Fig. 2). The highest summer air temperatures (25.3°C) increased the likelihood of growth anomalies of trees growing on Ordzhonikidze rock glacier by 2.9 times (95% confidence interval: 1.5 to 5.7 times) and on Turgen Aksu rock glacier by 4.6 times (2.0 to 10.6 times) compared to the lowest summer air temperatures (19.4°C). Weaker effects were found at temperatures below 25.3°C and at lags of one year or more (Fig. S9). No significant effects were found for Karakorum and Kugalan Tash rock glaciers, probably as a result of the smaller sample sizes (Tables S2 and S3 and Figs. S4–S7). The movements of the investigated rock glacier are also in line with annual mass balance of Tuyuksu glacier, which fluctuates asynchronously with rock glacier activity (Fig. 2). In other words, when Tuyuksu glacier loses mass, rock glacier movements are on the rise, thus confirming a link between summer air temperature and rock glacier activity in the Tien Shan. A correlation between rock glacier activity and seasonal snow cover duration and depth^21^,^22^ or with seismic activity^25^, as suggested by other authors, cannot be confirmed, although some years with early snowmelt coincide with high rock glacier activity in the 1960s.

**Figure 3:**
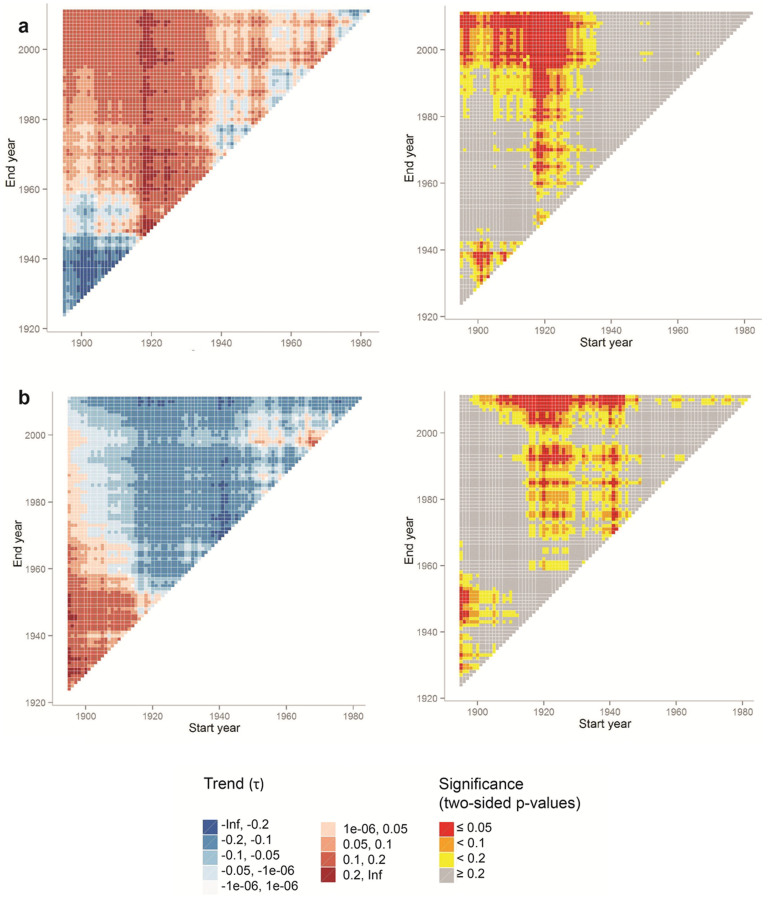
Contrasting activity trends of rock glaciers in the first (a, Karakorum) and second phase (b, Ordzhonikidze) of inactivation (1895–2011). Shown are Mann Kendall trend matrices with the standardized test statistic (τ) and significance levels (two-sided p-values) for different start and end years and a minimum period of 30 years.

**Figure 4:**
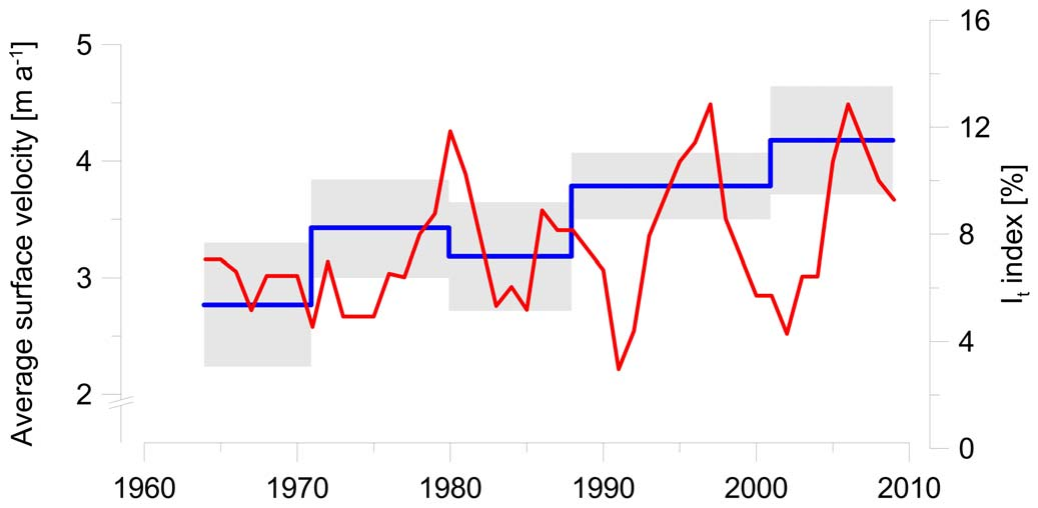
Activity of Karakorum rock glacier (1964–2009). 5-year-running-mean of I_t_ Index from dendrogeomorphic analysis (red) and average velocities in the lower part of the rock glacier from photogrammetric analysis (blue; 1σ confidence interval in grey).

At multi-decadal timescales, the rock glacier fronts under investigation exhibit contrasting trends (Figs. 3 and S10). The increase in air temperatures since the 1970s^26^ has most likely triggered increasing movement rates at Karakorum rock glacier, as evidenced by tree-ring records and photogrammetric displacements (Figs. 4 and S11), but no clear increases in movement rates (Figs. S12–14) or growth anomalies (Fig. S8) at the other sites, except for the upper part of Ordzhonikidze rock glacier (Fig. S13). These long-term trends most likely reflect the different phases of rock glacier response to the strong increase in air temperature, and illustrate the stabilization of rock glacier frontal zones due to topographic inactivation, insufficient debris supply or a reduction of the deformable ground ice content, or a combination thereof^19^.

The first phase (speed-up) applies to Karakorum rock glacier, where growth anomalies in trees have become increasingly frequent since the year 2000, with the highest activity assessed in 2007 (Fig. S4), and annual surface velocity rates are high. In the lowest part of the rock glacier, movement rates have significantly increased from 2.8 ± 0.5 m a^−1^ (1964–1971; mean values ±1 standard deviation) to 4.2 ± 0.5 m a^−1^ (2001–2009; Fig. S11). The steep frontal slope and the rapidly changing surface geometry of the rock glacier snout from compression further confirm that the lower part and the frontal scarp of Karakorum rock glacier have been speeding up after a surface rupture in summer 1985^10^.

The second phase (inactivation) has most likely already started on the lowest part of Ordzhonikidze and at Turgen Aksu and Kugalan Tash rock glaciers, where trees are recording decreasing amounts of growth anomalies (Figs. S5–8), in particular since the 1980s, and where horizontal velocity displacement rates in the lowest parts have shown no statistically significant change (Figs. S12–14). Velocity rates are considerably lower than at Karakorum rock glacier, with 0.8 ± 0.4 m a^−1^ on Ordzhonikidze (1966–2013), 0.4 ± 0.2 m a^−1^ on Turgen Aksu (1943–2012) and 1.4 ± 0.3 m a^−1^ on Kugalan Tash (1956–2012), respectively. The decreasing activity rates as from tree rings, or constantly low displacement rates as from image analysis, seem to be a combined effect of topographic inactivation – all investigated rock glaciers have reached the valley bottom –, of dynamic inactivation from insufficient debris supply, and of a shrinking ice core in the lower areas of the rock glaciers, where the lower limit of sporadic permafrost is reached^6^.

The different phases of rock glacier reaction to atmospheric warming occur simultaneously on Ordzhonikidze rock glacier (Fig. S13), where surface velocity has been steadily increasing since the 1950s in the uppermost – and thus coldest – part of the rock glacier, probably as a result of enhanced viscosity due to increasing ice temperatures and water percolation. In the middle part of Ordzhonikidze rock glacier, surface velocity has increased between the 1950s and the 1980s and is since then decreasing, probably as a result of the shrinking ice core and a consequently thickening active layer, which reduces the sensitivity to short-term variations in surface energy balance. Roughly constant displacement rates have been observed in the lowest part, where a new equilibrium with a reduced ice core on a flat bed-topography seems to have been established a while ago.

## Conclusion

The decadal-scale activity of the four observed rock glaciers is synchronous and reveals a positive association with summer air temperatures. Such a direct signal is remarkable in view of the rock glaciers' surface insulation by the rocky active layer and the thermal inertia of the ice core. These dampening effects are probably outweighed by the particularly large deformation sensitivity of ice-rock mixtures close to the melting point, with water being a potential coupling agent^14^,^23^.

On multi-decadal scales, the different trends of surface velocities among and within the rock glaciers reflect different site-specific conditions and phases of rock glacier response to the strong increase in air temperature, i.e. the stabilization of rock glacier frontal zones due to topographic inactivation, insufficient debris supply or a reduction of the deformable ground ice content, or a combination thereof^19^.

The implications on freshwater availability are ambivalent: On the one hand, the recent warming in Central Asia and the related melting of ice in the frontal parts of the investigated rock glaciers, as indicated by our study, implies that freshwater storage at the lower limit of sporadic permafrost in the Tien Shan is decreasing, as a thinner and deeper-lying ice core decreases the release of seasonal water. On the other hand, as all investigated rock glaciers terminate in rivers, the formation of rock-glacier dammed lakes, like Zhasyk Kol twenty kilometers upstream of Karakorum rock glacier, is probable – in particular for the accelerating Karakorum rock glacier, but potentially also for the other three advancing rock glaciers. Under continuing glacier shrinkage, these lakes could become alternative water reservoirs, but they also hold a flood danger, which adds to the hazard potential from degrading rock glaciers such as slope instability and debris flows^9^,^27^,^28^ as well as to the exacerbation of extreme flood peaks during thunderstorms when warm rain infiltrates into the frozen debris layer^8^.

## Methods

### Dendrogeomorphology

We followed standard dendrogeomorphic field methods^29^ to collect and prepare increment cores and cross-sections from 30 junipers (*Juniperus* sp.) and 250 Tien Shan spruces (*Picea* *shrenkiana* (Fish. & C.A. Mey.) subsp. *tianshanica* (Rupr.)) growing on or at the front of rock glaciers. As a rule, two cores or one cross-section were sampled from each tree. The LINTAB system (Rinntech) was used to measure ring widths, which were then cross-dated with a reference chronology collected from 30 undisturbed spruces growing in the immediate vicinity of Ordzhonikidze rock glacier. Kugalan Tash rock glacier was excluded from the regional signal analysis (Fig. S8) due to limited availability of trees for sampling. In a next step, the year and intensity of growth anomalies on all samples were identified and summarized per tree. Reactions occurring within 4 years on samples of the same tree were summarized and noted as one reaction in the year of first occurrence so as to account for reaction lags, which are common with e.g. growth decreases and compression wood.

### Statistical analysis

As a proxy for rock glacier activity, we then calculated the I_t_ index^15^ as the ratio between the number of trees showing a reaction (R_t_) and the total number of sampled trees (A_t_) on a rock glacier in any given year (t):
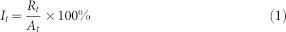
As climate data cover the past century and sample size is low before that time, we limited our analysis to the period 1895–2011. Periods of high and low activity were assessed from above- and below-average values of the 5-year running mean of the I_t_ indices^30^,^31^.

Long-term trends of rock glacier activity were analyzed with the two-sided non-parametric Mann-Kendall trend test at the 20, 10 and 5% significance levels^32^. With the help of moving time windows, the multiple trend tests were computed for the I_t_ indices of the investigated rock glaciers and for mass balance of Tuyuksu glacier for time windows of at least 30 years in length during the common 1895–2011 period.

Effects of summer (JJA) air temperature on rock glacier activity from 1895 to 2011 were quantified using distributed lag models^16^. Temporal lag effects were considered by shifting the series of summer temperature forward in time from zero to five years. For each rock glacier, we fitted a generalized linear model (GLM) with a binomial distribution as defined by the probability mass function:
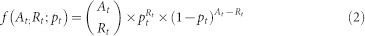


The probability p_t_ of a tree showing a reaction in year t was modelled with a logit-link function:
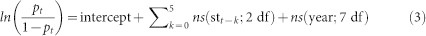
where ln is the natural logarithm and 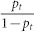 is the odds in year t. A natural cubic spline (ns) function with 2 degrees of freedom (df) allowed for non-linear relationships and was applied to summer temperature (st) lagged from 0 to 5 years. To control for long-term patterns of rock glacier activity due to e.g. topographic inactivation, the variable “year” was included in the model with an ns function with 7 df. These distributed lag non-linear models were fitted with functions included in the packages “dlnm”^33^ (version 2.0.6) and “splines” (version 3.0.2) from the statistical computing software R. Model output is presented as odds ratios, i.e. the ratio of the odds of a specific temperature to the odds of a reference temperature (here the lowest temperature measured in the series) is shown. The odds ratios are not affected by the scale of the predictor variable, e.g. standardizing the summer air temperature with the mean and the standard deviation does not change the odds ratios.

### Photogrammetry

In a first step, orthoimages with a spatial resolution of 0.5 to 2 m have been produced from the available Soviet era aerial photos and high-resolution satellite images (see SI). In a second step, displacements of trees, groups of trees, or other distinct visual features close to sampled trees were tracked within these stacks of co-registered orthoimages through image cross-correlation^17^,^18^, where image quality was sufficient, or manually digitized. In addition, distinct features close to the moving targets but on assumed stable ground outside of the rock glaciers were tracked to reference the moving targets and thus minimize the effects of residual co-registration deficiencies or distortions from inaccuracies of the ASTER GDEM, which was used for orthorectification. The accuracy of the displacements of each target was estimated as the mean offset of the displacement path from a straight line. This estimate is conservative as any real, and well expected, deviation from straight target motion is included in the error budget.

## Author Contributions

The study concept was developed by A.S. and M.S.; the dendrogeomorphic data were collected and processed by A.S. and A.R.; A.K. carried out the photogrammetric analysis and C.B. performed the statistical analysis. All authors were involved in the analysis, paper writing and revision process.

## Supplementary Material

Supplementary Information Supplementary Information

Supplementary Information Animation S1

## Figures and Tables

**Figure 1:**
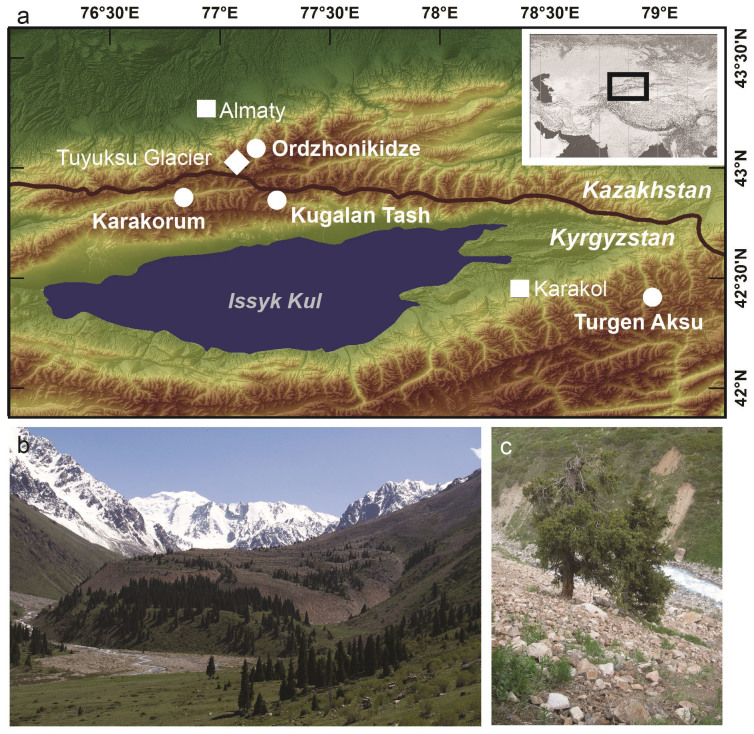
Overview of the study sites and sampled trees. (a). Location of Karakorum, Kugalan Tash, Ordzhonikidze and Turgen Aksu rock glaciers in the Kyrgyz and Kazakh parts of the Tien Shan. This map has been created with ArcMap Version 10.0 and is based on digital elevation information from the Shuttle Radar Topography Mission (http://srtm.csi.cgiar.org). (b). Forested terminus of Ordzhonikidze rock glacier. (c). Disturbed tree on the frontal slope of Ordzhonikidze rock glacier. Photographs taken by A. Sorg.
