# Supplementary material for: Contrasting responses of Central Asian rock glaciers to global warming
Source: Sci Rep. 2015 Feb 6;5:8228. doi: 10.1038/srep08228 (PMC4319170; doi:10.1038/srep08228)
Supplement: Supplementary Information [file srep08228-s1.pdf]

Contrasting responses of Central Asian rock glaciers to global warming

Supplementary Information

Annina Sorg*[1,2], Andreas Kääb[3], Andrea Roesch[2], Christof Bigler[4] and Markus Stoffel[1,2,5]

[1] Climatic Change and Climate Impacts Group, Institute for Environmental Sciences (ISE), University of Geneva, Route de Drize 7, CH-1227 Carouge, Switzerland

[2] dendrolab.ch, Institute of Geological Sciences, University of Berne, Baltzerstrasse 1+3, CH-3012 Berne, Switzerland

[3] Department of Geosciences, University of Oslo, P.O. Box 1047, N-0316 Oslo, Norway

[4] Forest Ecology, Institute of Terrestrial Ecosystems, Department of Environmental Systems Science, ETH Zurich, Universitätstrasse 16, CH-8092 Zürich, Switzerland

[5] Department of Earth Sciences, University of Geneva, Rue des Maraîchers 13, CH-1211 Geneva 4, Switzerland

1. Study sites

Many rock glaciers in the Tien Shan have developed during the Holocene; their formation is likely to be associated with high rates of debris accumulation during phases of glacier retreat[1]. All investigated rock glaciers are of (para-)glacial origin and emanate from cirques with small residual glaciers at altitudes between 3300 and 3800 m asl (Table S1), where they have been overwhelmed and depressed during Little Ice Age glacier advances[2]. The steep cirque walls and lateral slopes supply the debris for the rock glaciers[3], which consist mainly of weathering-prone coarse-crystalline granite, schists and, in the case of Karakorum rock glacier, Cambrian gneisses[1,3,4]. The steep terminal fronts (>37°; Fig. S1) with loose boulders and the "drunken trees" (Fig. S2) growing on the rock glaciers indicate that the investigated rock glaciers are highly active[4-6]:

2. Dendrogeomorphology

More than 40 years ago, the principles of dendrochronology were further developed to include the dating of geomorphic events, thus establishing the principles of dendrogeomorphology[7]. While the dendrogeomorphic approach has been widely applied in the field of natural hazards[8], the lack of trees at high elevations, namely in permafrost regions, has so far limited its application in rock glacier research to a few studies[9-12]. The principal idea behind the dendrogeomorphic approach in a periglacial context is that trees growing on rock glaciers will react to unstable growing conditions from pronounced rock glacier activity with the formation of growth anomalies. Horizontal forces in the shallow root zone can result in tree tilting ("drunken forest"[9]) and thus initiate the formation of compression wood, but also lead to the (partial) destruction of the root plate and thus induce a strong growth reduction. The burial of the tree stem base may lead to abrupt growth suppression as well. All these reactions can be dated with annual or even seasonal resolution.

3. Data

Climate data

Time series of temperature, precipitation and snow depth were available in daily resolution from Almaty station (43.23°N, 76.93°E, 851m asl, ECA station code 3289; Fig. 1) for the time period 1891-2011 (temperature and precipitation) and 1924-1995 (snow depth). The data were downloaded from the Royal Netherlands Meteorological Institute (KNMI) Climate Explorer (http://climexp.knmi.nl).

Mass balance

Mass balance has been assessed since 1957 at Tuyuksu glacier, which makes this data set the longest mass balance series in Central Asia. The mass balance data were made available through the World Glacier Monitoring Service[13], the original author is P. A. Cherkasov. For the period 1895-1957, we relied on reconstructed mass balance of Tuyuksu glacier[14-16].

Earthquakes

We analysed all documented earthquakes with a magnitude higher than 5 and with an epicenter within a 200 km radius around 43°N / 77°E since 1895. The data were downloaded from the U.S. Geological survey earthquake data base (www.earthquake.usgs.gov: Significant Earthquakes World Wide (NOAA) and Historical & Preliminary Data (PDE) catalogs).

Aerial photographs and satellite imagery

All aerial photographs used in this study were provided by the Institute of Geology of the National Academy of Sciences of the Kyrgyz Republic and the State Mapping and Geodetic Service of the Kyrgyz Republic. The Corona declassified satellite photos were obtained from the US Geological Survey (www.earthexplorer.usgs.gov). The contemporary commercial high-resolution satellite images stem from DigitalGlobe and their sensors Ikonos, Quickbird, GeoEye, WorldView-1 and WorldView-2. The availability of aerial photographs and satellite imagery is shown in Table S4.

Contemporary high-resolution satellite images with spatial resolutions between 0.5 to 1 m for all four sites were co-registered, if more than one was available for a site, and orthorectified using

the ASTER Global DEM (tests with Shuttle Radar Topography Mission DEM have also been performed) and UTM projection. Based on these master orthoimages, the Soviet era aerial photos and the 1970s Corona declassified satellite photos were oriented and rectified using rational function models based on 40-50 control points each. The resulting orthoimages have spatial resolutions of 0.5 to 2 m.

4. Supplementary tables

Table S1 ¦ Characteristics of the investigated rock glaciers.

	**Coordinates**	**Elevation (m asl)**	**Area (km^2)**	**Aspect**
Karakorum	42°52´N / 76°50´E	2650-3500	1.4	N
Kugalan Tash	47°46´N / 78°49´E	2970-3800	1.5	S
Ordzhonikidze	43°04´N / 77°09´E	2720-3500	2.9	NE
Turgen Aksu	42°25´N / 78°57´E	2800-3300	0.3	N

Table S2 ¦ Number of sampled Tien Shan spruces (*Picea shrenkiana* (Fish. & C.A. Mey.) subsp. *tianshanica* (Rupr.)) and junipers (*Juniperus* sp.) and dates of innermost tree ring of oldest trees on the investigated rock glaciers.

**Rock glacier**	***Picea shrenkiana***	***Juniperus sp.***	**Innermost year of oldest tree**
Karakorum	28	-	1856
Kugalan Tash	-	16	1687
Ordzhonikidze	180	-	1577
Turgen Aksu	73	4	1581

Table S3 ¦ Number of of growth anomalies on the investigated rock glaciers (percentages in brackets). trd: traumatic resin ducts; cw: compression wood; gs: growth suppression; gr: growth release; i: injury; ct: callous tissue.

Rock glacier	trd	cw	gs	gr	i	ct	total
Karakorum	9 (7)	14 (11)	78 (58)	31 (23)	-	1 (1)	133
Kugalan Tash	-	-	14 (45)	17 (55)	-	-	31
Ordzhonikidze	225 (25)	159 (18)	229 (27)	142 (16)	18 (2)	110 (12)	883
Turgen Aksu	32 (8)	11 (3)	208 (50)	151 (37)	2 (-)	8 (2)	412

Table S4 ¦ Aerial and satellite images of the investigated rock glaciers used for this study. A: aerial photograph, C: Corona satellite photo, S: contemporary satellite image.

Year	Karakorum	Kugalan Tash	Ordzhonikidze	Turgen Aksu
1943				A
1953			A	
1956		A		
1964	A			
1966			A	
1968				C
1971	C	C	C	
1973		A		
1976				A
1977	A			
1980	A	A		
1981				A
1985	A		A	
1988	A	A		
2001	S			S
2004		A		A
2008	S			
2009	S		S	
2012		S		S
2013			S	

5. Supplementary figures

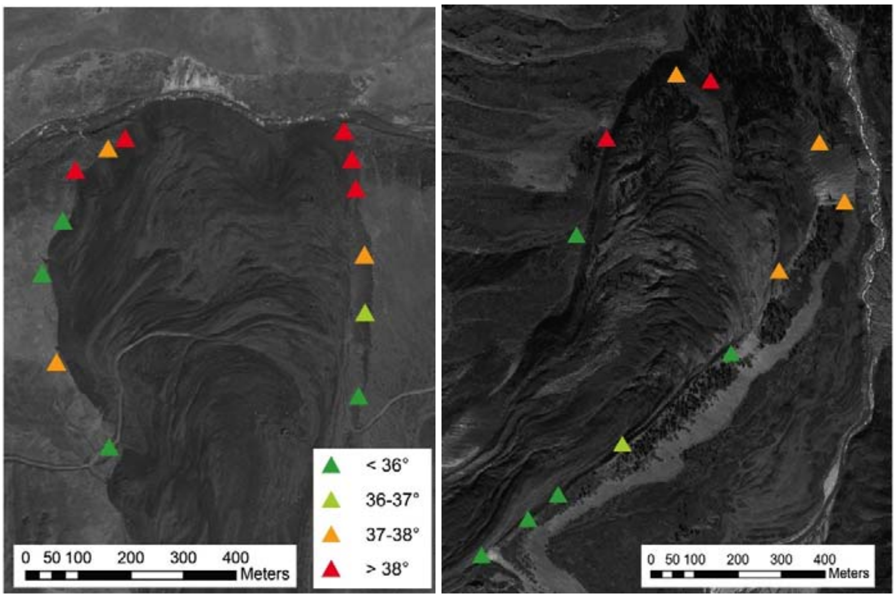

Fig. S1 ¦ Frontal slopes of Karakorum (left) and Ordzhonikidze rock glaciers (right).

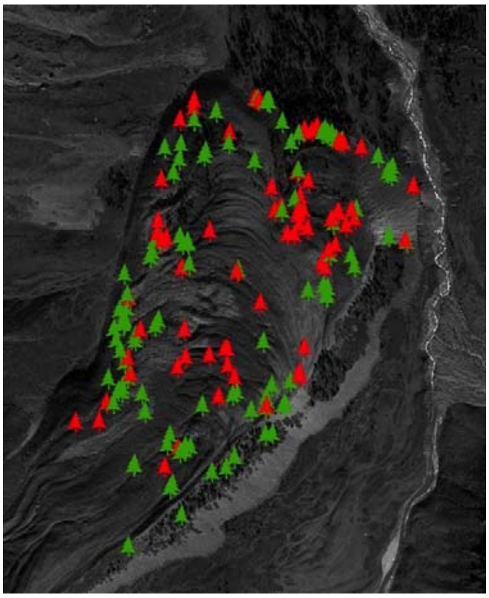

Fig. S2 ¦ Tilting direction of "drunken trees" on Ordzhonikidze rock glacier. Trees growing in the center of the rock glacier tend to be tilted backwards (upslope; red), whereas trees at the edge tend to bend away from the rock glacier (downslope; green).

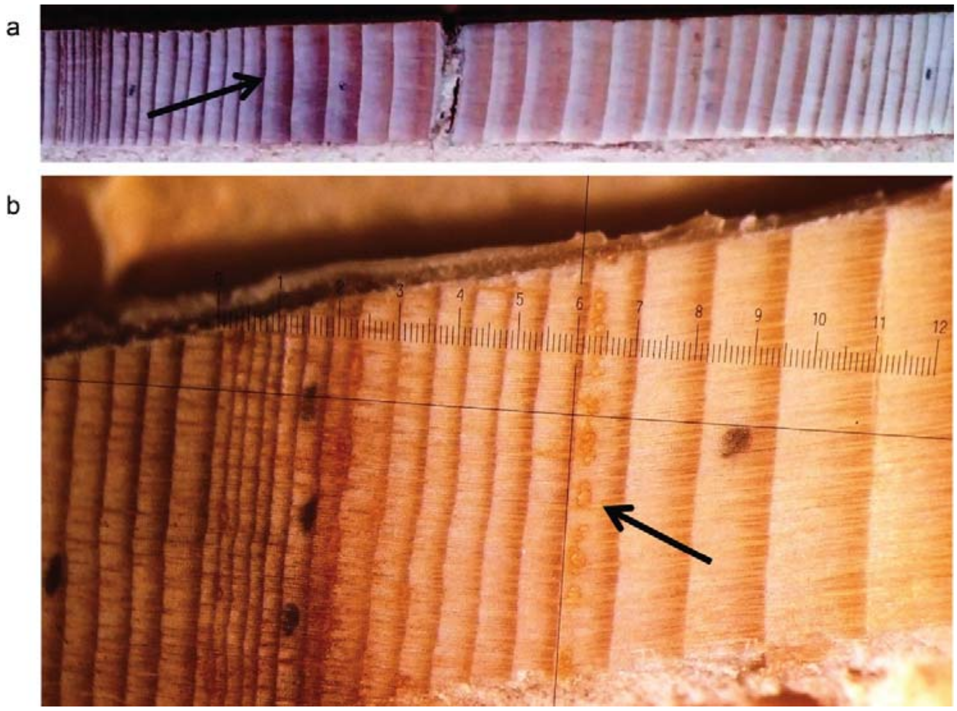

Fig. S3 ¦ Two typical reactions of disturbed trees on rock glaciers as observed on Ordzhonikidze rock glacier. **a**. Onset of compression wood after tilting of the tree. **b**. Formation of traumatic resin ducts after an injury from rockfall.

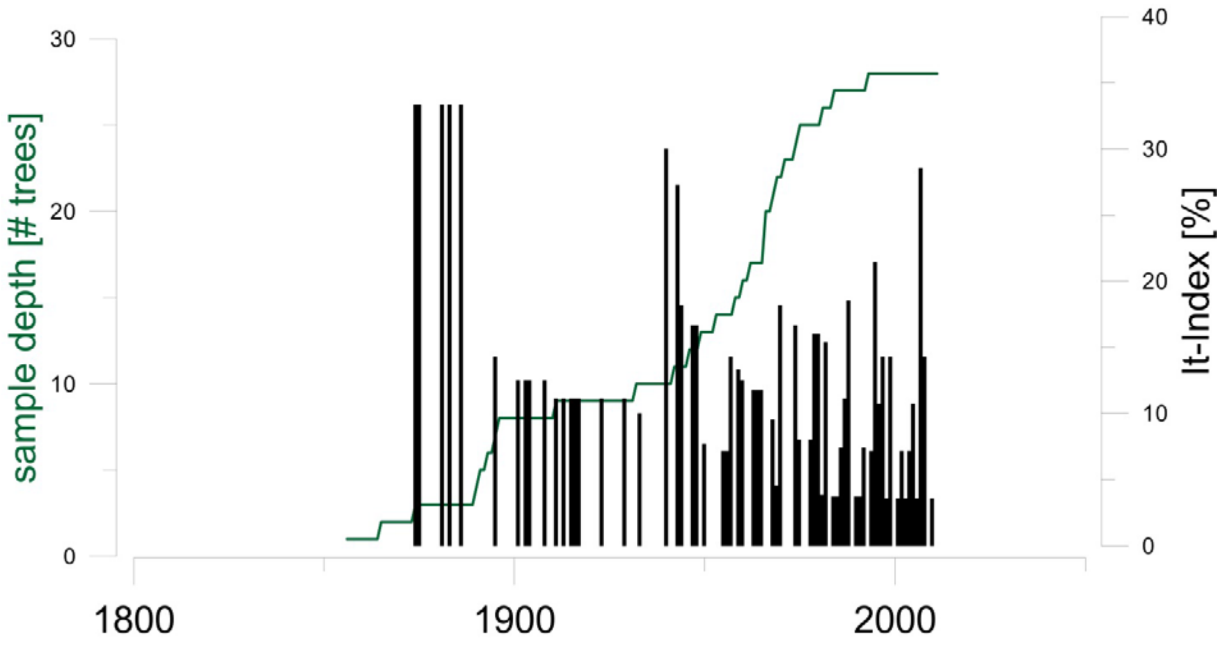

Fig. S4 ¦ Sample depth and I_t index for Karakorum rock glacier.

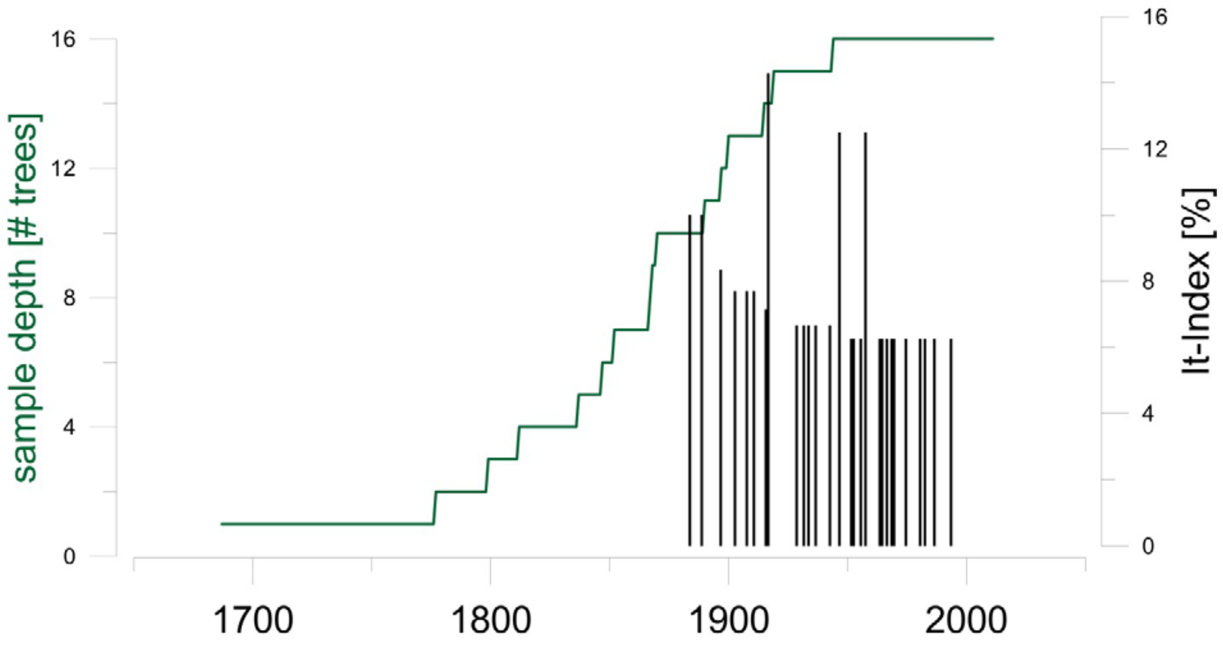

Fig. S5 ¦ Sample depth and I_t index for Kugalan Tash rock glacier.

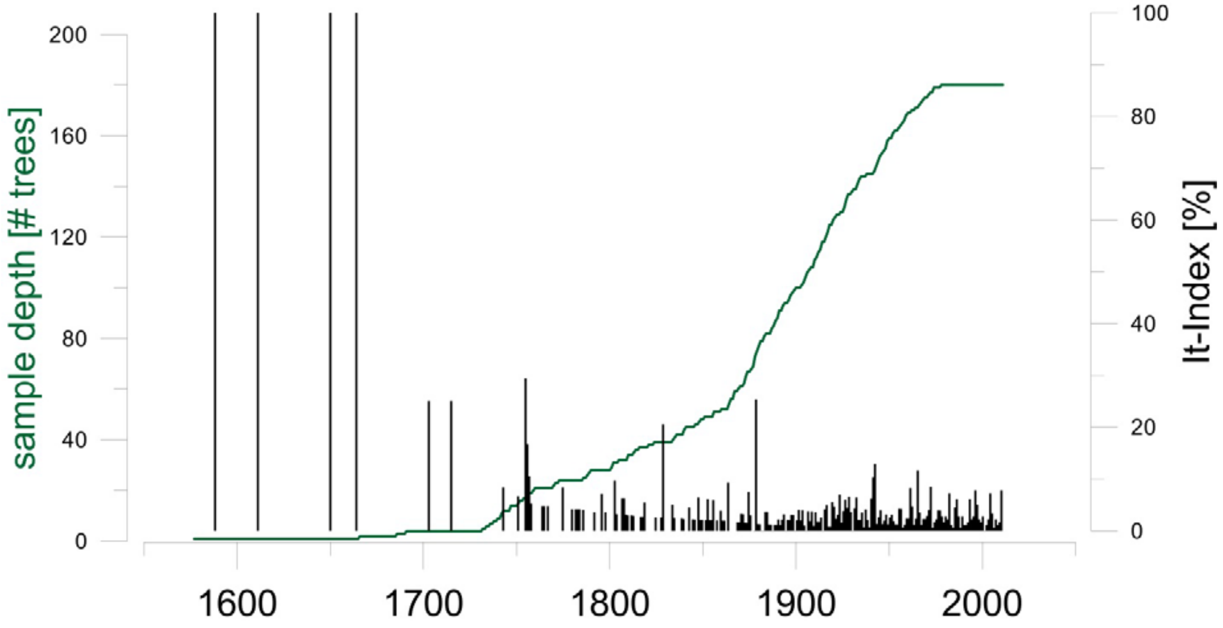

Fig. S6 ¦ Sample depth and I_t index for Ordzhonikidze rock glacier.

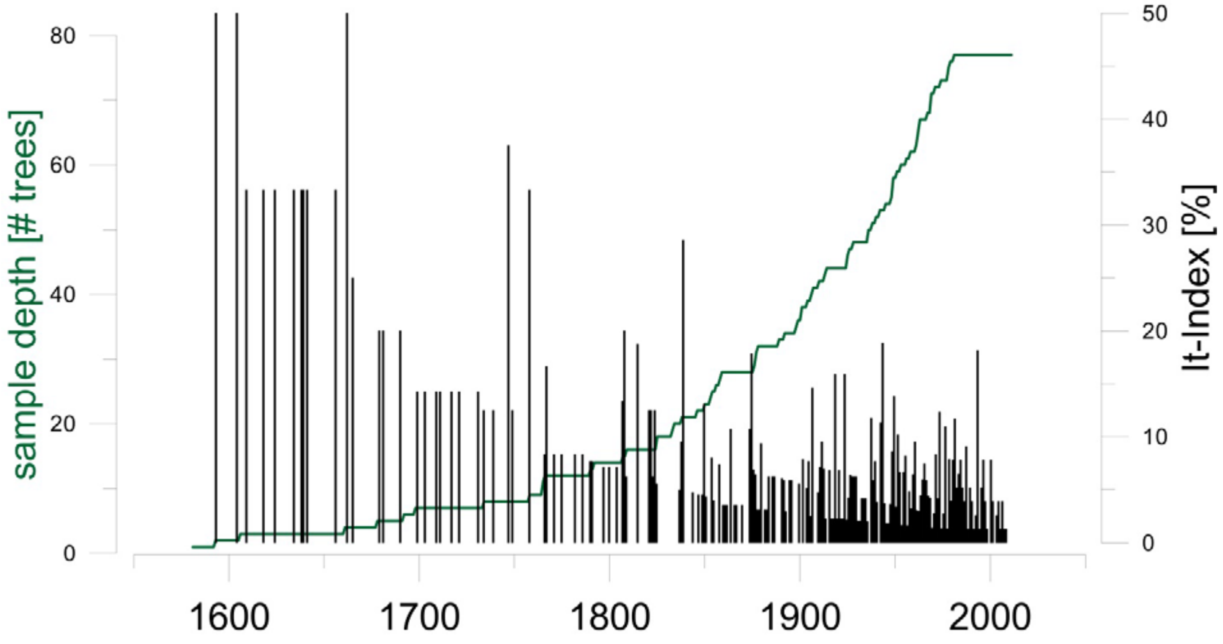

Fig. S7 ¦ Sample depth and I_t index for Turgen Aksu rock glacier.

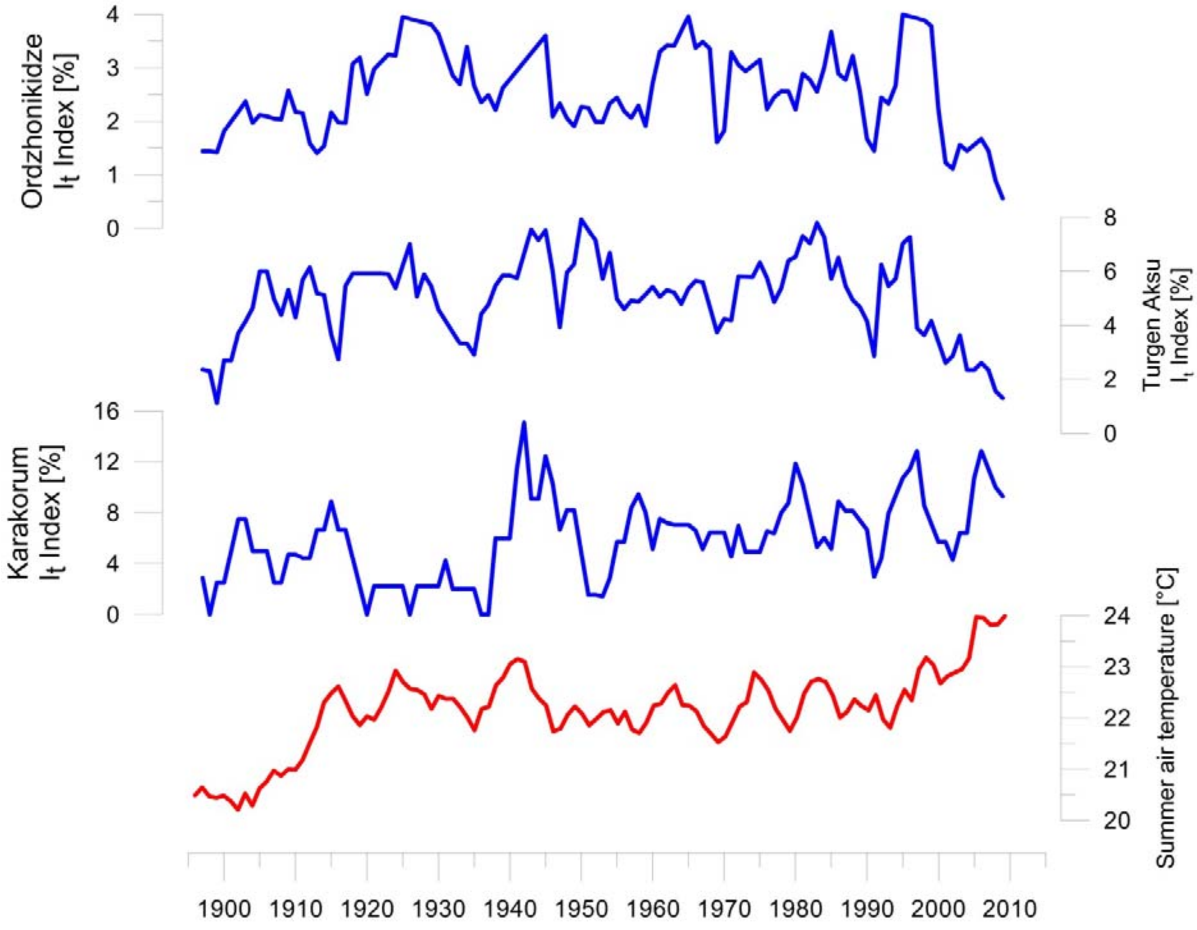

Fig. S8 ¦ I_t indices of, Ordzhonikidze, Turgen Aksu and Karakorum rock glaciers and summer air temperatures (JJA) in Almaty (1895-2011; 5-year-running-means). Kugalan rock glacier is not shown due to the limited sample size.

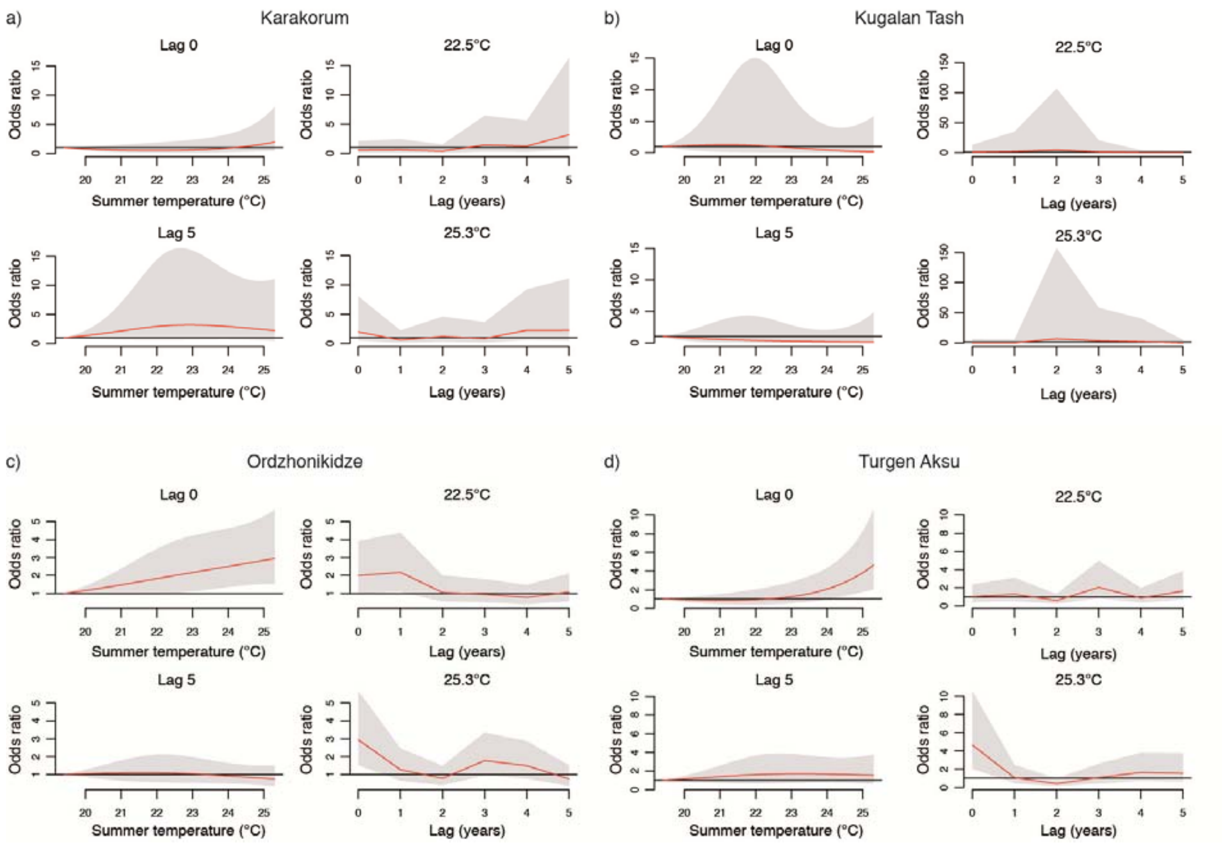

Fig. S9 ¦ Effects of summer temperature on rock glacier activity (1895-2011) shown for a) Karakorum; b) Kugalan Tash; c) Ordzhonikidze; and d) Turgen Aksu rock glaciers. On the left, odds ratios from the distributed lag models (equation 3; expected value: red line; 95% confidence interval: gray shading) are shown at fixed lags (0 and 5 years) and for varying summer temperatures. On the right, odds ratios for fixed summer temperatures (22.5°C and 25.3°C) and varying lags are shown. Odds ratios were calculated in comparison to the lowest summer temperature (19.4°C) observed in the series. Note the different scales of the y axes.

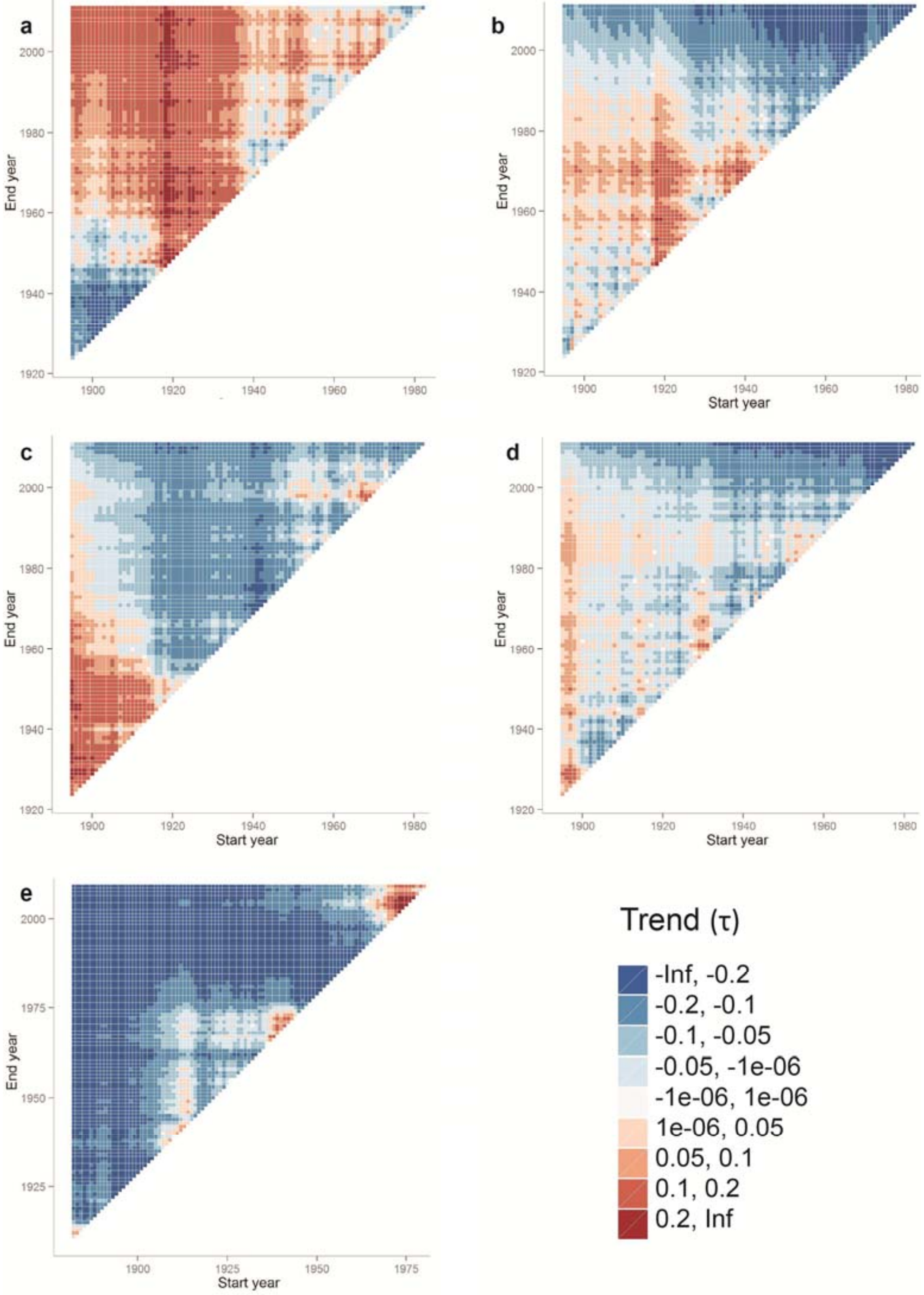

a
b
c
d
e
End year
Start year
Trend (τ)
-Inf, -0.2
-0.2, -0.1
-0.1, -0.05
-0.05, -1e-06
-1e-06, 1e-06
1e-06, 0.05
0.05, 0.1
0.1, 0.2
0.2, Inf

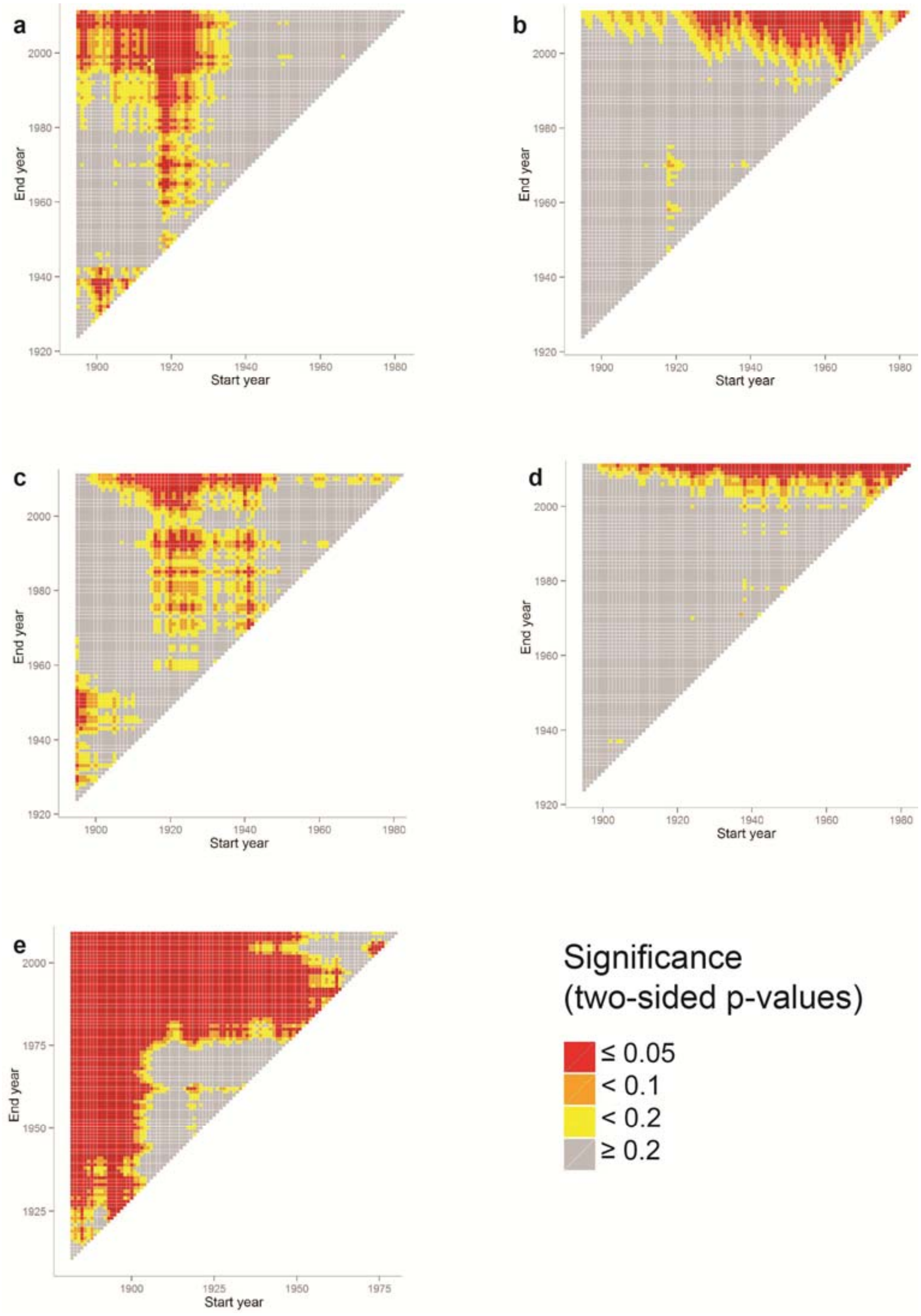

Fig. S10 ¦ Mann Kendall trend matrices of rock glacier- and glacier activity with the standardized test statistic (τ; above) and significance levels (two-sided p-values; below) for different start and end years and a minimum period of 30 years (1895-2011). **a.** I_t index Karakorum rock glacier. **b.** I_t index Kugalan Tash rock glacier. **c.** I_t index Ordzhonikidze rock glacier. **d.** I_t index Turgen Aksu rock glacier. **e.** Mass balance Tuyuksu glacier.

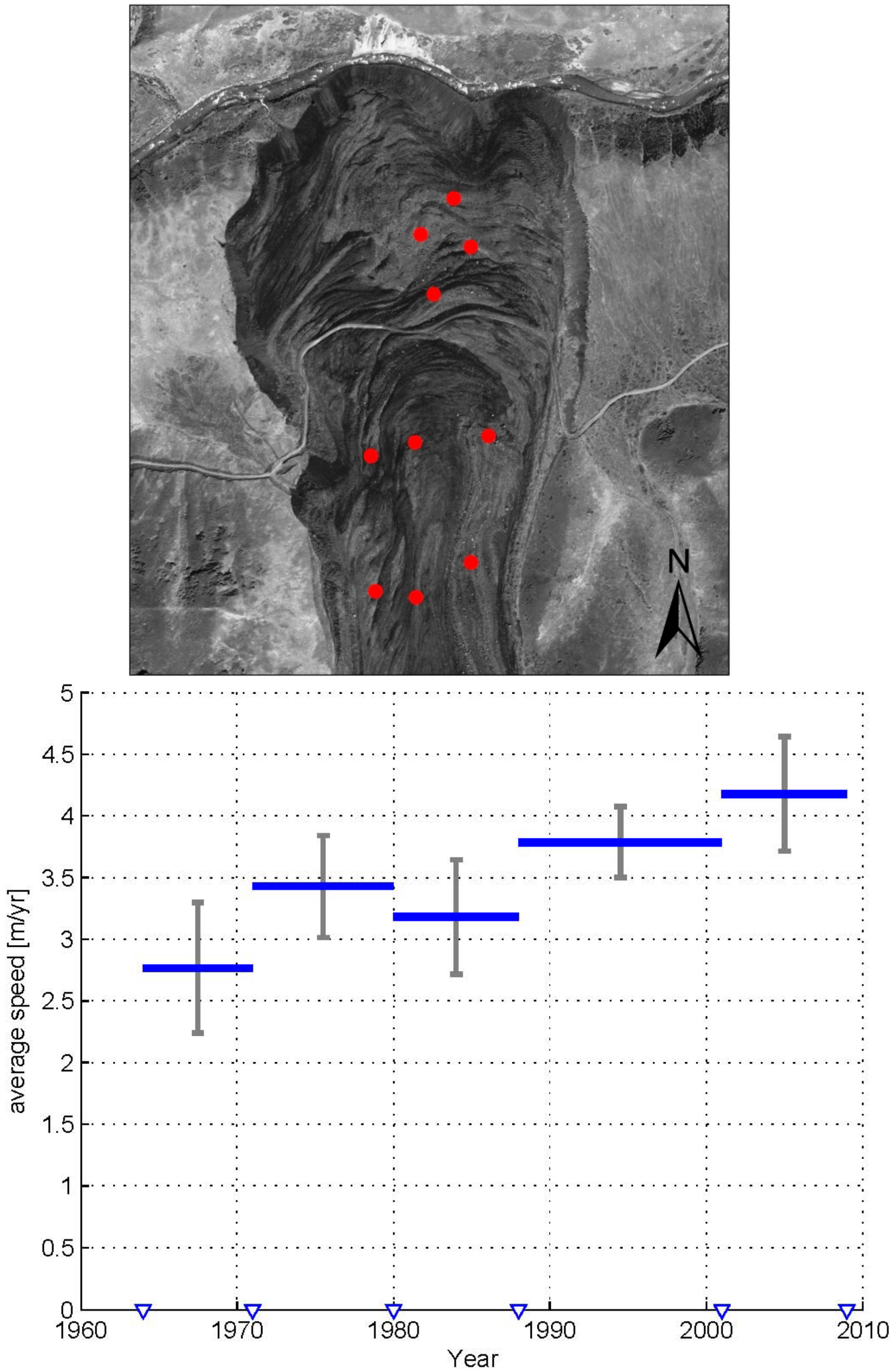

Fig. S11 ¦ Location of clusters used for photogrammetric analysis (above) and average speed with 1σ confidence interval (below) at Karakorum rock glacier.

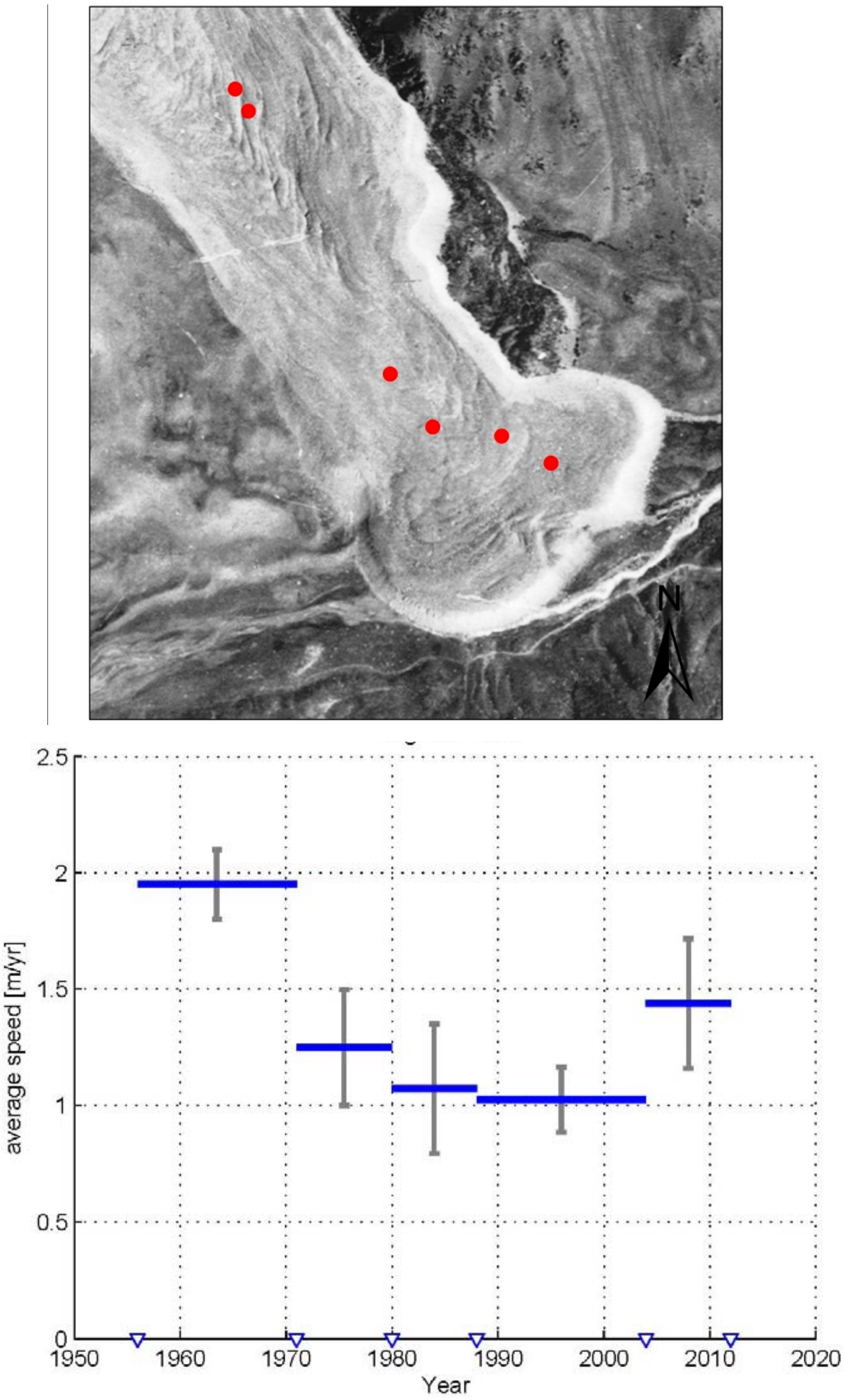

Fig. S12 ¦ Location of clusters used for photogrammetric analysis (above) and average speed with 1σ confidence interval (below) at Kugalan Tash rock glacier.

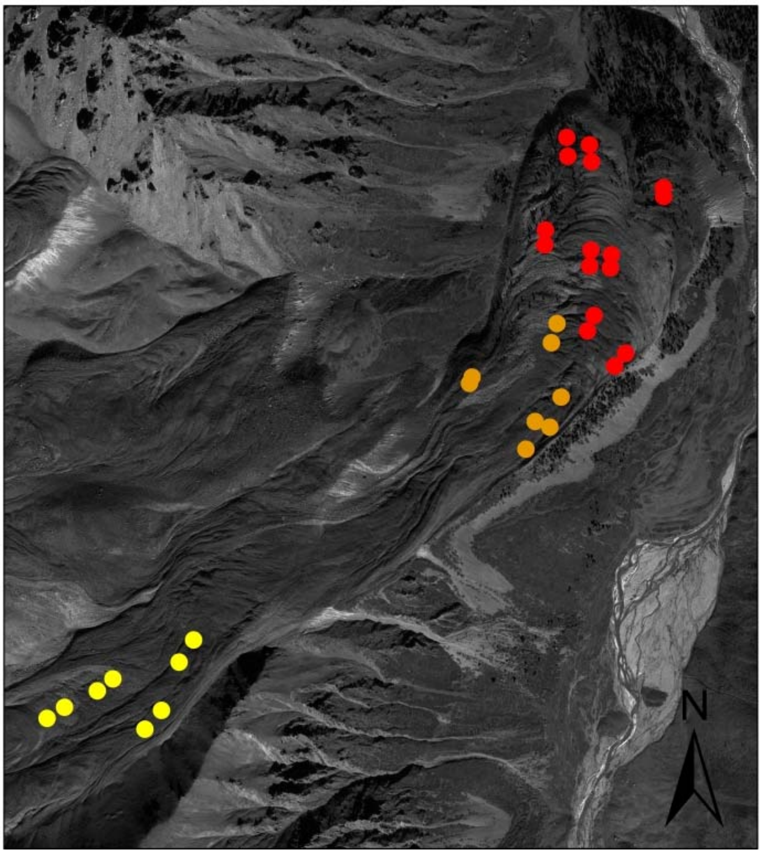
N

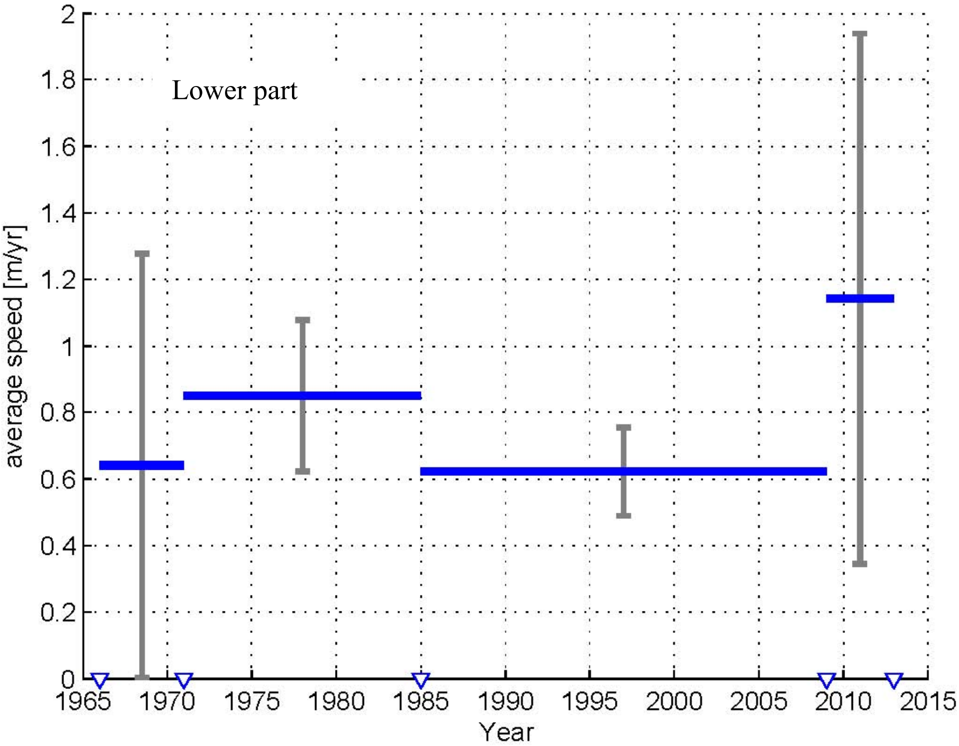
Lower part
average speed [m/yr]
0
0.2
0.4
0.6
0.8
1
1.2
1.4
1.6
1.8
2
1965
1970
1975
1980
1985
1990
1995
2000
2005
2010
2015
Year

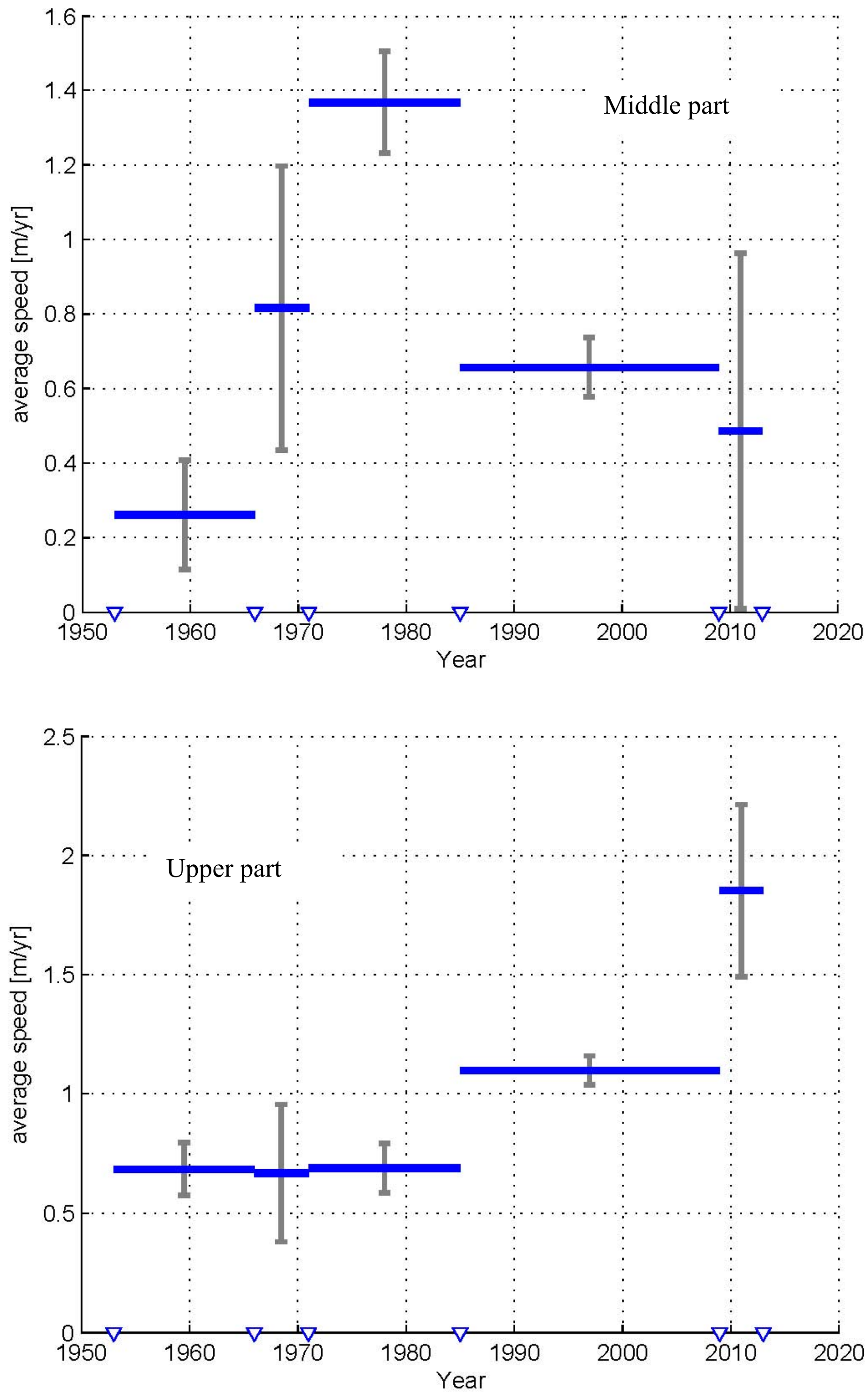

Fig. S13 ¦ Location of clusters used for photogrammetric analysis (above) and average speed with 1σ confidence interval (below) at Ordzhonikidze rock glacier. Three regions have been differentiated on the rock glacier: lower part (red), middle part (orange) and upper part (yellow).

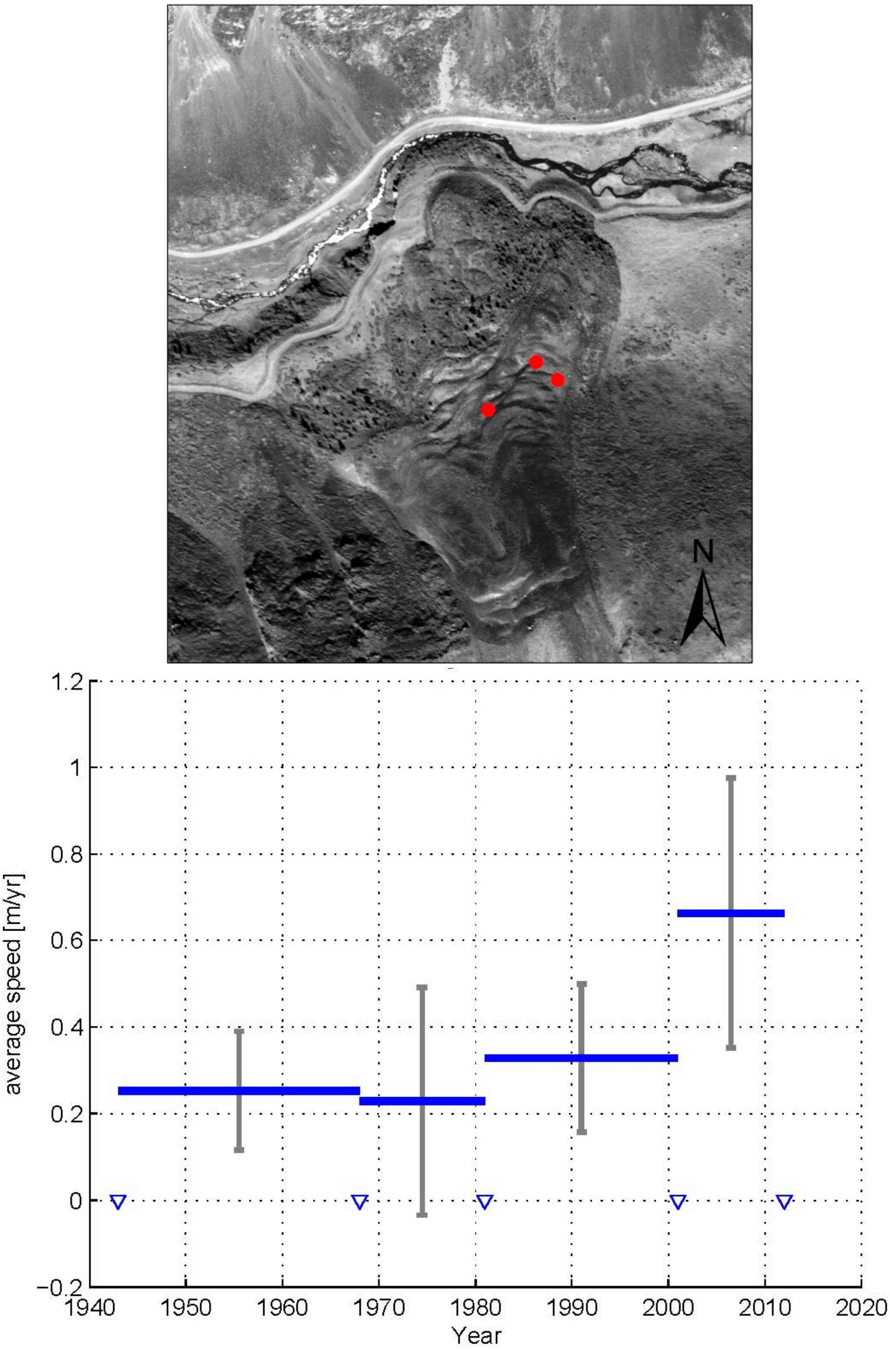

Fig. S14 ¦ Location of clusters used for photogrammetric analysis (above) and average speed with 1σ confidence interval (below) at Turgen Aksu rock glacier. Average speed is much lower than for the other rock glaciers and uncertainty is high due to bad image quality.

Supplementary animation

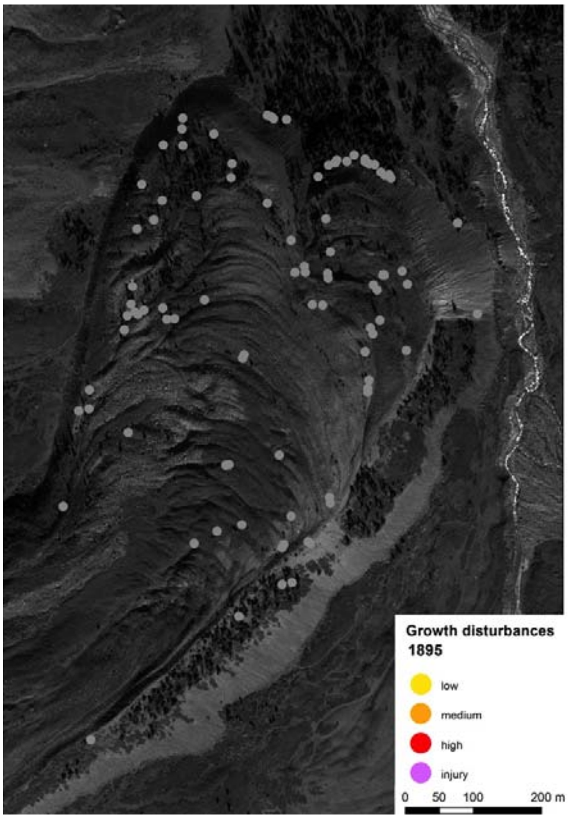

Animation S1 ¦ Animation of trees showing growth disturbances on Ordzhonikidze rock glacier during the period 1895-2011 (separate .avi-file).

References

1 Schröder, H., Kokarev, A. L. & Harrison, S. Rock glaciers in the northern Tien Shan, Kazakhstan: new data on movement rates and distribution. *Glacial Geology and Geomorphology* **1** (2005).

2 Gorbunov, A. P. Rock glaciers of the Zailyskiy Alatau (in Russian), in *Cryogenic phenomenons of Kazakhstan and Central Asia*. National Academy of Science SSSR, Yakutsk (1979).

3 Gorbunov, A. P., Titkov, S. N. & Polyakov, V. G. Dynamics of rock glaciers of the Northern Tien Shan and the Djungar Ala Tau, Kazakhstan. *Permafrost Periglac* **3**, 29-39 (1992).

4 Schröder, H. Aktive Blockgletscher im zentralen Teil des nördlichen Tienschan. *Petermanns Geographische Mitteilungen* **136**, 109-119 (1992).

5 Roer, I. & Nyenhuis, M. Rockglacier activity studies on a regional scale: comparison of geomorphological mapping and photogrammetric monitoring. *Earth Surf Processes* **32**, 1747-1758 (2007).

6 Barsch, D. *Rockglaciers. Indicators for the present and former geoecology in high mountain environments*. Springer, Berlin / Heidelberg, 331 (1996).

7 Alestalo, J. Dendrochronological interpretation of geomorphic processes. *Fennia* **105**, 1-140 (1971).

8 Stoffel, M., Bollschweiler, M., Butler, D. R. & Luckman, B. H. *Tree rings and natural hazards: A state-of-the-art*. Springer, Heidelberg, Berlin, New York, 505 (2010).

9 Zoltai, S. C. Tree ring record of soil movements on permafrost. *Arctic Alpine Res* **7**, 331-340 (1975).

10 Shroder, J. F. Dendrogeomorphological analysis of mass movement on Table Cliffs Plateau, Utah. *Quaternary Res* **9**, 168-185 (1978).

11 Giardino, J. R., Shroder, J. F. & Lawson, M. P. Tree-ring analysis of movement of a rock-glacier complex on Mount Mestas, Colorado, USA. *Arctic Alpine Res* **16**, 299-309 (1984).

12 Daanen, R. P., Grosse, G., Darrow, M. M., Hamilton, T. D. & Jones, B. M. Rapid movement of frozen debris-lobes: implications for permafrost degradation and slope

instability in the south-central Brooks Range, Alaska. *Nat Hazards Earth Sys* **12**, 1521-1537 (2012).

13 WGMS. Glacier Mass Balance Bulletin (2006-2007) *Glacier Mass Balance Bulletin* **10**, 96 (2009 and earlier volumes).

14 Dyurgerov, M. B., Uvarov, V. N. & Kostjashkina, T. E. Mass balance and runoff of Tuyuksu Glacier and the north slope of the Zailiyskiy Alatau range, Tien Shan. *Zeitschrift für Gletscherkunde und Glazialgeologie* **32**, 41-54 (1996).

15 Makarevich, K. G. Glaciation of the Zailisky Alatau (in Russian). *J. Rezul'taty Issledovaniy po Mezhdunarodnym Geojizicheskim Proyeklam. Glyatsiologiya* **23** (1969).

16 Mikhalenko, V. Changes in Eurasian glaciation during the past century: glacier mass balance and ice-core evidence. *Ann Glaciol* **24**, 283-287 (1997).
